# Spectro-spatial features in distributed human intracranial activity proactively encode peripheral metabolic activity

**DOI:** 10.1038/s41467-023-38253-7

**Published:** 2023-05-11

**Authors:** Yuhao Huang, Jeffrey B. Wang, Jonathon J. Parker, Rajat Shivacharan, Rayhan A. Lal, Casey H. Halpern

**Affiliations:** 1 Department of Neurosurgery, Stanford University Medical Center, Stanford, CA 94305 USA; 2 Medical Scientist Training Program, Stanford School of Medicine, Stanford, CA 94305 USA; 3 Department of Medicine (Endocrinology), Stanford University Medical Center, Stanford, CA 94305 USA; 4 Department of Pediatrics (Endocrinology), Stanford University Medical Center, Stanford, CA 94305 USA

**Keywords:** Feeding behaviour, Circadian rhythms and sleep, Metabolism

## Abstract

Mounting evidence demonstrates that the central nervous system (CNS) orchestrates glucose homeostasis by sensing glucose and modulating peripheral metabolism. Glucose responsive neuronal populations have been identified in the hypothalamus and several corticolimbic regions. However, how these CNS gluco-regulatory regions modulate peripheral glucose levels is not well understood. To better understand this process, we simultaneously measured interstitial glucose concentrations and local field potentials in 3 human subjects from cortical and subcortical regions, including the hypothalamus in one subject. Correlations between high frequency activity (HFA, 70–170 Hz) and peripheral glucose levels are found across multiple brain regions, notably in the hypothalamus, with correlation magnitude modulated by sleep-wake cycles, circadian coupling, and hypothalamic connectivity. Correlations are further present between non-circadian (ultradian) HFA and glucose levels which are higher during awake periods. Spectro-spatial features of neural activity enable decoding of peripheral glucose levels both in the present and up to hours in the future. Our findings demonstrate proactive encoding of homeostatic glucose dynamics by the CNS.

## Abstract (web summary)

How human brain activity relates to peripheral metabolism is not known. Here, the authors find that intracranial activity is strongly coupled to peripheral glucose variations across multiple brain regions and is sufficient for decoding of glucose levels.

## Introduction

Maintenance of peripheral glucose levels represents one of the most vital homeostatic control loops. The central nervous system (CNS) is heavily reliant glucose as a fuel, as it has the highest energy demand in the body^1^. As such, there is a teleologic basis to hypothesize that the CNS closely surveils and regulates body glucose levels. Growing evidence suggests the presence of distributed CNS ‘glucose-responsive’ neuronal populations, which respond either directly through glucose sensing or indirectly as a part of the glucose-modulatory circuit^2–4^. The most well-studied location of these neurons is the hypothalamus, whereby multiple nuclei, including the arcuate, paraventricular, ventromedial and lateral nuclei, have demonstrated effector functions that alter peripheral glucose levels^4–7^. The amygdalohippocampal complex (AHC) has also been shown to harbor these neurons^8–10^ and recently, the rodent hippocampus was reported to exert a modulatory effect on peripheral glucose level changes through sharp wave-ripples (SPW-Rs)^9^. Further, the habenular nucleus has been implicated in regulation of glucose metabolism, as lesion in this area increased insulin sensitivity^11^. Other direct glucose-sensing neurons have also been found in the nucleus accumbens^12^, the thalamus^13^, and the prefrontal cortex^14^.

In humans, direct evidence supporting intracranial regulation of peripheral metabolism remains limited. Thus far, non-invasive functional imaging studies coupled with an insulin or glucose challenge in humans have implicated various corticolimbic regions such as the insula and orbitofrontal cortex as potential regulators of glucose homeostasis^15,16^. Further support for an anatomically distributed brain network involved in glucose regulation comes in part via coupling of spectral power measured by scalp EEG with glucose fluctuations^17^. However, a longitudinal human (i.e., over the course of days) study simultaneously assessing large-scale neuronal activity across multiple brain regions and peripheral glucose levels has not been performed.

Here, we provide an in-human study combining longitudinal intracranial recordings with continuous glucose monitoring (CGM), a similar technology used in previous CNS-glucose studies^9,17,18^. Given known presence of glucose-responsive neuronal populations in the hypothalamus and across multiple corticolimbic structures, we hypothesize that distributed CNS population neural activity encodes information regarding ongoing and future energy demand that are reflected in peripheral glucose dynamics. To test this hypothesis, we obtained intracranial electrographic recordings from corticolimbic structures alongside time-synchronized interstitial glucose concentrations using a CGM device over a multi-day period. In one subject, we had the unique opportunity to directly record from the ventral diencephalon including the hypothalamus.

First, we found that HFA in the bilateral hypothalami was strongly correlated with peripheral glucose variations in a diurnally mediated fashion. Across several corticolimbic sites, we also observed significant HFA-glucose correlations that were diurnally mediated. Second, to understand the source of correlation, we performed wavelet coherence analysis and identified strong circadian coupling across corticolimbic areas between HFA and peripheral glucose variations, which was partly explained by hypothalamic connectivity. Removal of circadian influence in the signals revealed significant but diminished correlations in non-circadian (ultradian) HFA and glucose variations. This correlation of ultradian rhythms in HFA and glucose variations was in turn higher during wakeful periods. Finally, we trained a multivariate decoder using spectral profiles of all gray matter recordings to predict peripheral glucose variations. This model was able to proactively decode glucose levels both in the present and hours in advance using circadian and ultradian signal dynamics. Taken together, these results suggest CNS activity may be a leading indicator of glucose dynamics.

## Results

### Study participants

**Fig. 1:**
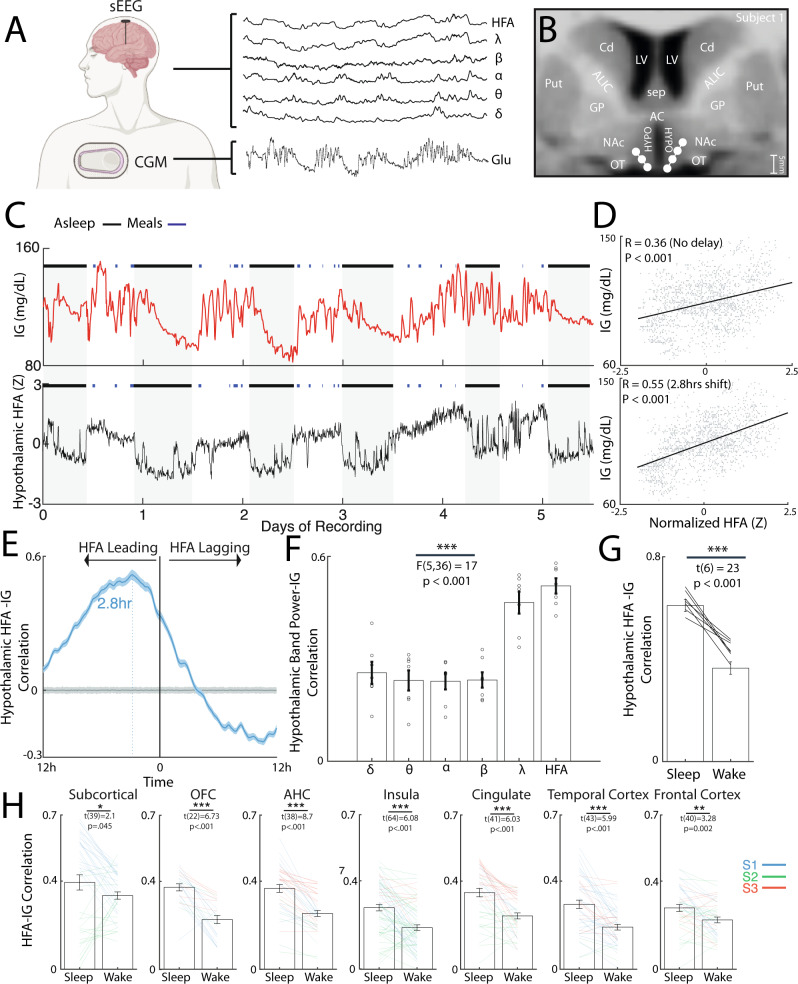
Human hypothalamic and corticolimbic activity is correlated with interstitial glucose dynamics in a diurnally mediated fashion. **A** Clinical recording system comprising stereo-encephalography (sEEG) and continuous glucose monitoring (Dexcom G6) in patients undergoing invasive monitoring. This illustration was created with BioRender.com **B** Anatomical location of electrodes in the bilateral hypothalamus of Subject 1. **C** Interstitial glucose levels as a function of time over six recording days in Subject 1 with corresponding mean high-frequency activity (HFA; 70–170 Hz) from the hypothalamic electrodes. The black lines denote periods of sleep while the blue lines denote start and end of ad libitum meals. **D** Scatter plots of hypothalamic HFA and interstitial glucose variations with 0-h (top) and −2.8 h lags (bottom). Pearson’s correlation (R) and associated *P*-value are shown. *N* = 1588 datapoints. **E** Mean cross-correlogram between hypothalamic HFA and interstitial glucose variations. The error bar indicates the standard error. Cross-correlograms using shuffled glucose data with 2 standard deviations is shown in gray. *N* = 1588 datapoints. **F** Mean lag-corrected correlation between hypothalamic activity and interstitial glucose variations across conventional powerband (*N* = 7 hypothalamic channels, one-way ANOVA; F(5,36) = 17, *P* < 0.001). **G** Lag-corrected hypothalamic HFA-glucose correlation stratified by sleep and wake states (*N* = 7 hypothalamic channels, one-sample T-test: t(6) = 23, *P* < 0.001). **H** Subcortical and corticolimbic lag-corrected HFA-glucose correlation stratified by sleep and wake states across three subjects. All tests were Paired t-test with statistics and *P*-value shown per region. All error bars indicate standard error of the mean (SEM). Source data are provided as a Source data file. *P < 0.05, **P < 0.01, ***P < 0.001.

Three subjects were included in the study, and all had simultaneous coverage of hippocampus, amygdala, insula, cingulate, orbitofrontal cortex, and frontal and temporal cortices (Supplementary Table 1). Continuous peripheral glucose measurements were recorded in conjunction with time-synced intracranial activity (Fig. 1A). Subject 1 (S1) had two depth electrodes targeted at bilateral hypothalami. The number of days with continuous glucose monitoring and intracranial recordings were 5.5, 6.5, 9.3 for S1, S2, and Subject (S3), respectively. The sleep chronotype for each subject demonstrated distinct sleep/wake cycles (Supplementary Fig. 1).

### Coupling of hypothalamic and corticolimbic activity to peripheral glucose variations is diurnally mediated

Given the established role of hypothalamus in both sensing and regulating peripheral glucose levels, we first examined the relationship between hypothalamic HFA and peripheral glucose variations in S1 (Fig. 1B–G). Glucose concentrations in the interstitial fluid was continuously monitored alongside HFA (Fig. 1C) while the subject had ad libitum meals and self-determined sleep-wake cycle. Mean hypothalamic HFA was significantly correlated to interstitial glucose variations with no lag correction (Fig. 1D: R = 0.36, *N* = 7 hypothalamic channels). Higher correlation was found by using HFA as a leading indicator and coupling it with future interstitial glucose levels (R = 0.55, temporal lag: 2.8 h). This was visualized using a cross-correlogram which showed overall higher hypothalamic HFA correlation to interstitial glucose levels when HFA has a leading temporal shift (Fig. 1E). Across conventional powerbands, hypothalamic HFA was associated with the highest correlation (Fig. 1F: one-way ANOVA: F(5,36) = 17, *P* < 0.001). To evaluate if the correlation was driven purely by day-night cycling, we stratified the time-series by sleep and wake periods. The hypothalamic HFA-glucose correlation was significantly higher during sleep periods (Fig. 1G: Paired t-test: t(6) = 23, *P* < 0.001). Having observed hypothalamic HFA is strongly correlated to peripheral glucose dynamics, we asked if the broader subcortical and corticolimbic regional activity also reflected glucose variations. Across three subjects, mean regional lag-corrected HFA-glucose correlation ranged from 0.23 to 0.38 with subcortical regions exhibiting the highest mean correlation (Supplementary Fig. 2A: one-way ANOVA: F(6,287) = 13, *P* < 0.001). When evaluating the distribution of temporal lag accounting for the highest correlation across all regions, we observed high intra- and inter-individual variability, with median being −1.8, −1.2, and +7.3 h, respectively, for subject 1, 2, and 3 (Supplementary Fig. 2B). In a similar fashion to hypothalamic HFA-glucose coupling, mean correlation between HFA and interstitial glucose variations was higher during periods of sleep across subcortical and corticolimbic regions (Fig. 1H, Paired t-test: all *P* < 0.05). As there have been studies associating interictal epileptiform discharges (IEDs) to glucose levels^19^, we evaluated the extent to which IEDs were correlated to glucose levels in our dataset. In Subject 1 and Subject 3 with epilepsy, we found that IEDs in the seizure onset zone had lag-corrected correlation 0.22 and 0.20 with interstitial glucose variations, respectively (Supplementary Fig. 3). We also evaluated the relationship between hypothalamic HFA and the derivative of glucose levels (i.e., rate of change) (Supplementary Fig. 4). Hypothalamic HFA showed significant but modest correlation with glucose rate of change (R = 0.31) and was highest at a slight lagging temporal shift (5 min). This correlation was also modulated by powerbands with HFA exhibiting the highest correlation (Supplementary Fig. 4B: one-way ANOVA: F(5,36) = 15, *P* < 0.001). Across three subjects, mean regional lag-corrected HFA-glucose rate of change correlation ranged from 0.16 to 0.22 (Supplementary Fig. 4C).

### Circadian rhythm contributes to coupling between intracranial HFA and interstitial glucose dynamics

**Fig. 2:**
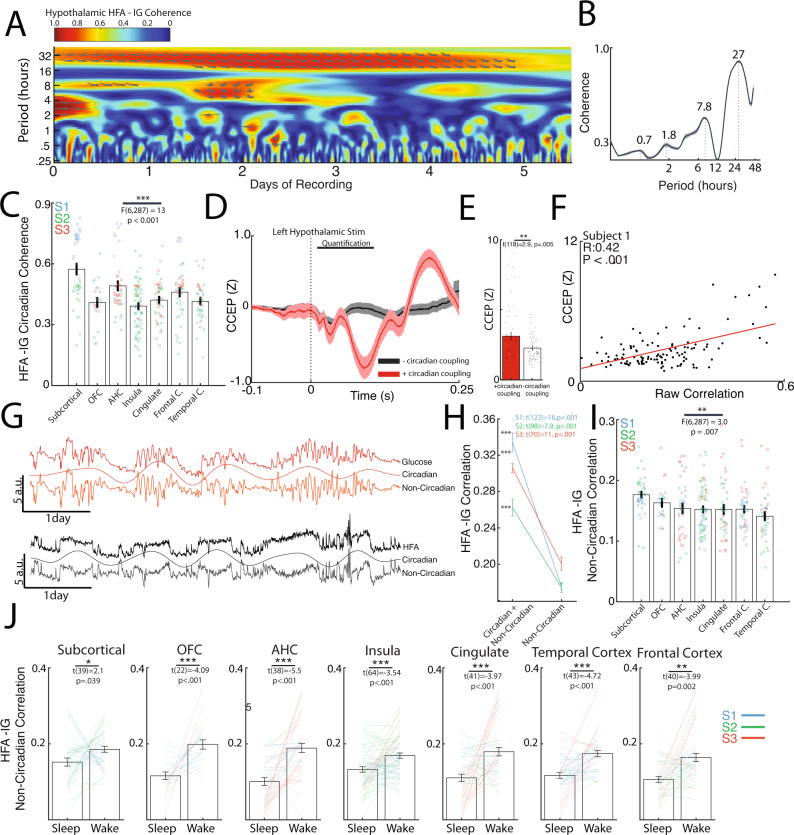
Circadian and ultradian rhythms underlie coupling of intracranial activity and interstitial glucose dynamics. **A** Wavelet coherence spectrum between hypothalamic HFA and interstitial glucose levels revealing high coherence at circadian periodicity. Arrows denote phase lag. **B** Corresponding coherence periodogram showing peaks in ultradian and circadian periodicity. **C** Circadian coherence between HFA and interstitial glucose across subcortical and corticolimbic regions (Nsubj = 3, Nchannels = 288, one-way ANOVA; F(6,287) = 13, *P* < 0.001). **D** Average cortico-cortical evoked potentials (CCEP) elicited by left hypothalamic stimulation in S1 stratified by whether channels’ HFA were significantly coupled to glucose in a circadian manner. Channels with significant glucose coupling showed an overall larger CCEP with left hypothalamic stimulation. Shaded error bars indicate error of the mean (SEM). **E** Average CCEP magnitude (0.01–0.1 s) was significantly higher in channels with significant circadian coherence to glucose (two-sample t-test, t(118) = 2.9, *P* < 0.001). **F** Left hypothalamic CCEP magnitude correlates directly with HFA-glucose lag-corrected correlation across all channels in S1 (Nchannels = 121, Pearson’s R = 0.42, *P* < 0.001). **G** Wavelet decomposition of circadian and non-circadian rhythms in interstitial glucose dynamics and mean hypothalamic HFA (Subject 1). Combining the circadian and non-circadian rhythms reconstruct the original signal trace. **H** Removal of circadian contribution to HFA and interstitial glucose variations resulted in significant reduction of correlation across 3 subjects (Paired t-test with statistic shown for each subject). **I** Lag-corrected non-circadian correlation between HFA and interstitial glucose across subcortical and corticolimbic regions (Nsubj = 3, Nchannels = 288, one-way ANOVA; F(6,287) = 3, *P* = 0.007). **J** Subcortical and corticolimbic lag-corrected non-circadian HFA-glucose correlation stratified by sleep and wake states across three subjects (Nchannels per region is noted by the degree of freedom, all tests were Paired t-test with statistics and *P*-value shown per region). All error bars indicate SEM. Source data are provided as a Source data file. *P < 0.05, **P < 0.01, ***P < 0.001.

To understand the timescales of correlation underlying HFA and interstitial glucose variations, we evaluated the mean hypothalamic HFA-glucose wavelet coherence spectrum (Fig. 2A). This revealed high coherence at circadian periodicity, with a constant HFA-glucose phase lag. The corresponding spectral periodogram (Fig. 2B) showed peaks in ultradian (0.7, 1.8, 7.8 h) and circadian periodicities (27 h). The spectral periodogram for glucose rate of change also exhibited multiple ultradian (1.6, 4, 6, and 10 h) and circadian periodicities (20 h, Supplementary Fig. 4D). The mean circadian coherence varied by regions ranging from 0.39 to 0.57 with subcortical, amygdalohippocampal and frontal regions exhibiting the highest magnitudes (Fig. 2C: one-way ANOVA: F(6,287) = 13, *P* < 0.001). Across all gray matter electrodes, the mean circadian coherence was 0.49, 0.40, and 0.44 with a mean leading HFA phase lag of 1.8, 9.5, and 7.3 h for Subjects 1, 2, and 3, respectively (Supplementary Fig. 2C). In channels with significant circadian coherence, the HFA-glucose circadian phase lag was linearly associated with the correlational temporal lag (Supplementary Fig. 2D: Pearson’s correlation, R = 0.36, *P* < 0.001), indicating that the circadian phase lag as a contributing source to the correlational temporal lag. We conducted a secondary analysis evaluating circadian coherence using a narrow circadian window (20–28 h vs 16–36 h; Supplementary Fig. 5A, B). This revealed similar circadian coherence values with similar regional trend in mean values, indicating circadian coherence did substantially depend on the circadian window employed.

In Subject 1, CCEP mapping allowed for interrogation of functional connectivity amongst brain regions with high HFA-glucose coherence. Specifically, channels with significant HFA circadian coupling to glucose levels had larger CCEP waveforms when the left hypothalamus was stimulated, implying that these regions are functionally connected to the hypothalamus (Fig. 2D). Accordingly, the mean CCEP amplitude was significantly higher in channels with circadian coupling (Fig. 2E; two-sample t-test, t(118) = 2.9, *P* = 0.005). The same was observed with right hypothalamic stimulation (Supplementary Fig. 6A, B; two-sample t-test, t(121) = 3.6, *P* < 0.001). Further, the magnitude of raw correlation across regions correlated with mean CCEP (Fig. 2F, Pearson’s correlation, R = 0.42, *P* < 0.001), which was present with right-sided stimulation (Supplementary Fig. 6C, Pearson’s correlation, R = 0.42, *P* < 0.001).

### Ultradian rhythm underlies correlation between intracranial HFA and interstitial glucose dynamics during wakeful periods

To evaluate if circadian coupling accounted for the HFA-glucose correlations entirely or if there was coupling on faster timescales, wavelet decomposition was used to separate the raw signals into its circadian (18–36 h) and ultradian components (0–18 h) (Fig. 2G). Removal of circadian influence to the HFA and interstitial glucose time series reduced mean correlation by 52%, 35%, and 41%, respectively, for S1, S2, and S3, respectively (Fig. 2H: S1 mean correlation: 0.31 to 0.15, S2 mean correlation: 0.23 to 0.15, S3 mean correlation: 0.29 to 0.17, Paired t-test: all *P* < 0.001). Across subcortical and corticolimbic regions, the ultradian correlation ranged 0.14 to 0.18, with subcortical regions having the highest magnitude of correlation (Fig. 2I: one-way ANOVA: F(6,287) = 3.0, *P* = 0.007). When stratified by sleep and wake cycles, ultradian correlation across regions showed significantly higher magnitude during wakeful periods than compared to sleeping periods (Fig. 2J, Paired t-test; all *P* < 0.05). Finally, as recent studies in non-diabetic patients have implicated meal-context and post-prandial interstitial glucose changes are related to appetite, changes in hunger level, and overall energy intake^20,21^, we compared ultradian correlation between HFA-glucose during pre-prandial and prandial periods. These analyses revealed variable direction of magnitude change based on region (Supplementary Fig. 7). Specifically, HFA-glucose ultradian correlation was decreased during prandial periods in majority of insula channels (Paired t-test: t(64) = 4.7, *P* < 0.001), whereas it was increased in the AHC and frontotemporal cortical channels (Paired t-test: all *P* < 0.05). Within individual subcortical structures, we also observed different sleep and meal-related changes in strength of both raw and ultradian HFA-glucose correlations (Supplementary Fig. 8A–C).

### Spatially variant spectral features of intracranial activity proactively decodes glucose levels

**Fig. 3:**
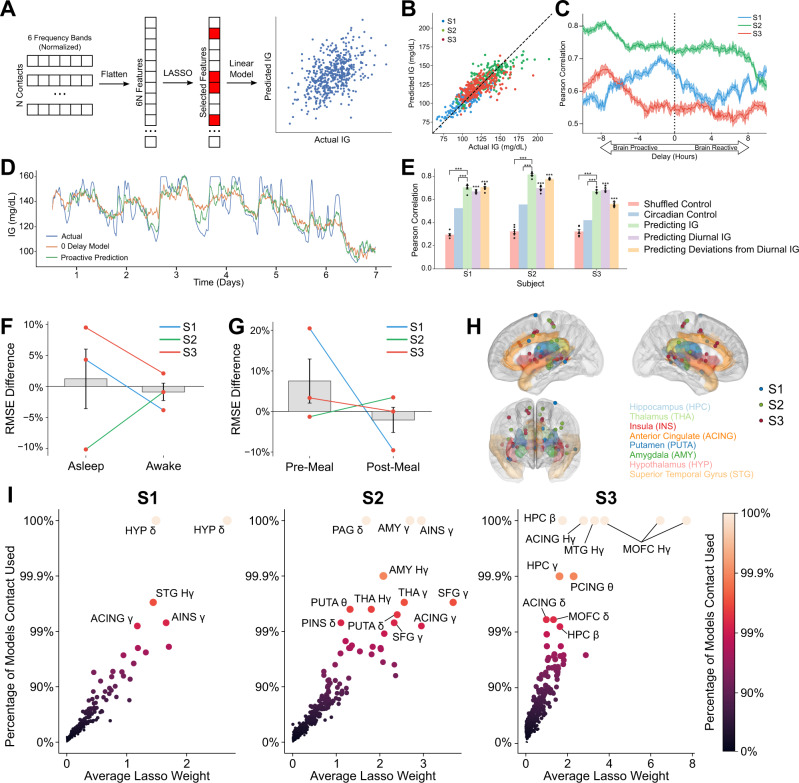
Human intracranial activity proactively decodes peripheral glucose at a fidelity beyond simple circadian dynamics. **A** Schematic of LASSO for interstitial glucose decoding. First, all 6 frequency bands across our N electrodes for each subject are flattened into a single feature vector. LASSO selects a subset of these features for regularization, and then trains a linear model to predict the interstitial glucose level. **B** Decoder performance for predicting interstitial glucose from iEEG oscillations. Different colored dots represent three separate subjects. **C** Model performance, as quantified by average Pearson Correlation between the actual and predicted interstitial glucose during 5-fold cross validation, given different temporal shifts between the iEEG data and interstitial glucose. Negative shifts indicate current iEEG data leading in prediction of interstitial glucose in the future (i.e., proactively). Shaded regions are ±1 SEM (*N* = 5 folds). **D** Moving-average time-trace of actual interstitial glucose (blue), predicted interstitial glucose given no temporal delay (orange), and proactively predicted interstitial glucose (green). **E** Cross-validated model performance given several conditions, with optimal temporal shifts. Error bars are ±1 SEM. *** indicates *p* < 0.001 when compared to shuffled control (*N* = 5 folds of cross-validation, two-sample Student’s T-test, exact *P*-values of all comparisons given in Source data file). **F** Percent difference in RMSE when compared to RMSE for all datapoints for all decoded interstitial glucose levels when asleep and awake (Nsubj = 3, two-sample Student’s T-test, *p* = 0.68). **G** Percent difference in RMSE when compared to RMSE for all datapoints for all decoded interstitial glucose levels preprandial and prandial. Comparison is insignificant (Nsubj = 3, two-sample Student’s T-test, *p* = 0.55). **H** Plots of contacts (denoted as circles) that contributed to at least 99% of LASSO models with randomized training and test sets in our three subjects. Individual subjects are given in Supplemental Fig. 11. **I** Scatterplot of model coefficients and how often LASSO selects this feature. Labeled datapoints appeared in at least 99% of models. Source data are provided as a Source data file.

Given the significant correlation between distributed intracranial activity and glucose levels, we sought to design a decoder for predicting glucose levels over time (Fig. 3A). We used LASSO regression, a machine learning method that identifies a subset of most important features and subsequently fits a linear regression model using reduced the set of features. This reduces the risk of overfitting given many features compared to a relatively limited number of datapoints. In this case, the feature space for this model included all power band activity (delta, theta, alpha, beta, gamma, and HFA) across gray matter contacts on a single subject level. Using this model, we found that we were able to accurately predict blood glucose across a wide range of glucose levels for each subject (Fig. 3B; Pearson’s Coefficient, average R = 0.73, *P* < 0.001). This model was repeatedly trained at consecutive time intervals to account for temporal lag and revealed that the model performance was highest when the intracranial features were hours prior to the glucose recording (Fig. 3C; temporal lag with highest decoding performance was 1.5, 7.5, and 7.5 h with powerband features leading for Subjects 1, 2, and 3, respectively). Compared to the zero-delay model, the proactive decoder captured faster variation in glucose variations in addition to the general temporal trend (Fig. 3D, Subject 2).

To validate that the glucose decoder is not simply relying on the coupled circadian rhythm of glucose and intracranial activity, we tested the decoder in several conditions (Fig. 3E). First, as a negative control, we found that decoder performance declines significantly when predicting glucose levels shuffled in 1-h windows. Second, using average glucose at a given time of day as a circadian control, we found this model performed better than chance, but was significantly inferior to the decoder trained on intracranial data (two-sample t-test, 0.50 vs 0.73, *P* < 0.001). Finally, we found that the decoder performance did not decline when using intracranial data to predict solely circadian glucose dynamics or non-circadian glucose levels, indicating that the decoder considers contributions from both circadian and ultradian rhythms in glucose levels over time. As a secondary analysis, we evaluated decoding for the derivative of interstitial glucose (Supplementary Fig. 9A). Modest decoding performance was only achieved in Subject 1.

To assess whether decoder fidelity varied across sleep cycle, we evaluated our decoder’s root mean square error (RMSE) across both wakeful and asleep periods of the day. We found that the percent difference in RMSE during wakeful vs asleep periods of the day compared to RMSE during the entire day was not significantly different (Figs. 3F, 31.2% vs −0.8%, Paired t-test, t(2) = 0.87, *P* > 0.05). In addition, we calculated the RMSE between actual and decoded glucose levels during sleep stages (N1, N2, N3, and REM) as scored by the SleepSEEG algorithm^22^. This calculated RMSE was compared to overall RMSE of the decoder and we found no significant difference in decoding performance (Supplementary Fig. 10A, B, one-way ANOVA, F(4,2) = 1.5, *P* > 0.26). Similarly, RMSE was not significantly different during pre-prandial vs prandial periods (Figs. 3G, 4.4% vs −1.9%, Paired t-test, t(2) = 0.93, *P* > 0.05).

To identify electrode channels selected by the LASSO-based glucose decoder, we performed a boot-strap Monte Carlo analysis. We found that across the 3 patients, a distributed set of corticolimbic structures were consistently used for LASSO regression (Fig. 3H, I). Across multiple training-test set permutations, there was a strong correlation between the number of permutations a specific channel was used in the decoder and the median linear weight in the decoder model, which can be interpreted as the strength of contribution of an electrode to the model (Fig. 3I). Commonly used structures that were shared across subjects include the insula and cingulate cortex (Fig. 3H, Supplementary Fig. 11). Notably, the hypothalamic contacts were consistently used in Subject 1 for decoding of both absolute glucose levels and glucose rate of change (Supplementary Fig. 9B).

## Discussion

Mounting evidence supports that the CNS can directly influence peripheral metabolic activity through not only regulation of caloric intake, but also causal glucose modulatory mechanisms^18,23–25^. This is thought to be achieved through distributed presence of glucose-responsive neurons in the hypothalamus as well as other corticolimbic regions^2–6,26^. However, how longitudinal CNS activity in humans relates to peripheral glucose variations has not been examined previously. No multi-day recordings of simultaneous CNS activity and glucose levels are currently available to allow study of physiologic rhythms in these signals. We address these questions through simultaneous sampling of putative glucose-responsive regions and tracking of peripheral glucose variations in humans. This dataset also gives us the unique potential to examine longitudinal changes in neurologic and metabolic activity over several days. Furthermore, we can examine a wide range of subcortical and cortical regions simultaneously, which is normally more difficult to accomplish in rodents and other small animal models.

As the hypothalamus is well implicated in the control of glucose metabolism, we first evaluated hypothalamic activity in relation to interstitial glucose variation. We identified high correlation between hypothalamic HFA and peripheral glucose dynamics, especially when corrected for a leading HFA temporal lag. To evaluate if this correlation was driven by the on and off nature of sleep, we calculated the correlation solely during sleeping or wakeful periods. This revealed a diurnally mediated pattern whereby the HFA-glucose correlation was significantly higher during periods of sleep not only in the hypothalamus, but also across corticolimbic regions. This may reflect a consequence of the robust nocturnal regulation of blood glucose, which is influenced by changes in secretion of hypothalamic and pituitary hormones^27,28^. Further, to understand the underlying rhythms contributing to HFA-glucose correlation, we used wavelet decomposition to uncover period-specific coupling. We found that circadian coherence was prominent not only in the hypothalamus, but also in channels across corticolimbic regions. This circadian coupling contributed to a large extent the correlation between the HFA and interstitial glucose variations, as suppressing the circadian component of the signal significantly decreased correlation. This was not surprising as the hypothalamus, through the suprachiasmatic nucleus (SCN), has been found to be necessary for diurnal rhythms in plasma glucose concentration^29^, independent of feeding patterns^30,31^. In addition, the circadian phase lag predicted the correlation temporal lag, indicating that the circadian component influences both magnitude and the temporal lag of HFA-glucose coupling. The circadian phase lag between regional HFA and glucose levels were also highly uniform across corticolimbic regions, manifesting as HFA circadian signal several hours ahead of the glucose circadian signal. These findings indicate that pervasive circadian coupling may be driven by a central circadian oscillator. To support this, in S1 with hypothalamic recording and CCEP mapping, we found that the magnitude of circadian glucose coupling in other regions was related to their hypothalamic effective connectivity. This suggests that variation in strength of glucose coupling on a regional basis is at least partly explained by functional connection to the hypothalamus. This was not surprising as the hypothalamus, through the suprachiasmatic nucleus, has wide projections and afferents from cortical and subcortical regions^32,33^. However, future work will be needed to probe whether brain regions with circadian glucose coupling arise from endogenous sensing mechanisms or driven by hypothalamic or other regional inputs. In addition, further work is needed to evaluate the variance in regional correlation strength and temporal lag. Although we found that the circadian phase shift accounted partly for the temporal lag seen in the HFA-glucose relationship, other factors such as sleep chronotype^34^, which was distinct amongst the three subject subjects, are also contributive. Future studies employing regulated sleep/wake cycle and/or constant routine paradigm will be needed to fully understand the temporal lag observed between brain activity and peripheral metabolism, as well as contribution from circadian versus sleep-related modulation.

As we initial observed multiple time scales of HFA-glucose coherence, we note that even after removal of the circadian component, multiple regions remained significantly correlated with peripheral glucose dynamics. This correlation may reflect a combination of ultradian regulation of gluco-regulatory hormones^35,36^, meal intake patterns^37^, and adaptive neural responses to glucose^2^. Notably, the ultradian correlation between HFA and glucose across regions was higher during waking periods, as compared to the original correlation being higher during sleeping periods. This suggests circadian physiology may predominant during sleep, thus driving correlation between brain activity and glucose, whereas physiologic events during wakefulness may enhance coupling on ultradian timescales (i.e., in the period of time around meals).

Given the distributed correlated activity between intracranial HFA and glucose variations, we sought to build and test a regression-based decoder to predict glucose levels using intracranial spectral activity as features. The decoder reported here accurately predicted glucose variations across three subjects, notably performing at a high fidelity for predicting both current glucose and proactively several hours into the future given current intracranial activity. This indicates that current intracranial activity can be used to decode both current and future glucose variations. This is in alignment with the finding that circadian coupling, which explains a high proportion of the correlation to glucose, has a proactive phase offset of several hours. As such, data realigned according to the circadian phase offset might have higher prediction accuracy. Mechanistically, the phase offset of hypothalamic activity and glucose variations suggests there may be several hours of delay in transmission of circadian CNS output to changes in peripheral metabolism. Prior studies have found the suprachiasmatic nucleus regulates hepatic glucose production and uptake either directly through the autonomic nervous system or indirectly through orexin^38–40^. However, we also found that decoding results could not simply be explained by circadian variations, with the full model performing significantly better than a pure diurnal model. This again highlights that intracranial activity likely couples to glucose on multiple timescales to include circadian, ultradian, and meal-related fluctuations. From a homeostatic perspective, it is possible to speculate based on these findings that the CNS may principally acts as a proactive controller of peripheral metabolism, governing glucose dynamics hours in advance through complex orchestration of feeding, hunger, satiety as well as peripheral glucose uptake and release^41–43^.

Predictive capabilities of blood glucose levels hours holds potential for augmenting current closed-loop insulin controllers for diabetes management, if our findings hold true for diabetic patients^44,45^. One of the disadvantages of current implementation of closed-loop controllers (and sliding-scale insulin regimens used inpatient) is that they are reactive to currently measured glucose. Playing “catch-up” with boluses of fast-acting insulin is typically inferior to basal-bolus regimens which use a combination of long-acting basal insulin with correctional boluses of fast-acting insulin^46^. However, optimal dosing of long-acting basal insulin takes weeks of close follow-up^47,48^. This makes it difficult to achieve optimal glucose control in the outpatient setting and impractical in the inpatient setting. Being able to predict glucose proactively hours in advance, as demonstrated here, could potentially enable the use of long-acting basal insulin in closed-loop controllers to achieve tighter glucose control. Future studies in patients with diabetes mellitus will be needed to evaluate if similar findings observed here can be replicated when glucose homeostasis is already disturbed.

### Limitations and future directions

Limited subjects are enrolled in this study given the difficulty and rarity of simultaneously recording multiple brain regions and continuous glucose over at least several days. The inclusion criteria required at least five days of monitoring to allow longer physiologic rhythms to be quantified (e.g., circadian rhythm), multi-site coverage of hypothesized gluco-regulatory regions and agreement to CGM implantation. These criteria resulted in a limited number of subjects, especially in the setting of COVID-19 pandemic. In addition, direct hypothalamic recording is rare given few clinical indications so only one subject had hypothalamic coverage. On a broader note, because recording locations are solely dictated by the clinical need for epilepsy localization, our electrode coverage varies from patient to patient. This makes it infeasible to use models trained on one subject to predict interstitial glucose from another patient. An interesting future direction would be investigating whether models trained on standardized scalp EEG could be used to predict glucose levels across a wide range of subjects. In addition, the use of CGM has not been formally FDA-approved for non-clinical use in patients without diabetes. However, the Dexcom G6 system has been successfully used in for non-clinical contexts, including for patients without diabetes to track post-prandial glucose dynamics^20,49^. Finally, our study design does not allow for direct causal interpretation, and as such intracranial activity and peripheral glucose variations might be correlated due to a potential third unmeasured variable. Future study employing direct electrical stimulation to activate or inhibit specific brain regions over varying time scales with continuous glucose monitoring may help dissect causal relationships.

Taken together, these results provide compelling evidence that peripheral glucose variations can be decoded from multi-site intracranial activity over days in a proactive manner. The basis of this decoding involves not only circadian coupling but also correlation on ultradian dynamics. Future blood glucose management strategies may thus involve sampling intracranial activity as a modality to enable proactive and tighter control of glucose dynamics.

## Methods

### Participants and inclusion criteria

Three human subjects (two females) who were observed in an epilepsy monitoring unit for clinical mapping met the inclusion criteria for this study from January 2020 to January 2021. The inclusion criteria included (1) simultaneous coverage of hippocampus, amygdala, insula, and orbitofrontal cortex (regions likely to harbor glucose-responsive neurons and are frequently targeted for epilepsy mapping), (2) no known history of diabetes mellitus, and (3) at least 5 days of continuous monitoring. Subject 1 had electrodes traversing the bilateral hypothalamic region given clinical concern of gelastic seizures arising from the hypothalamus in the absence of radiographic evidence of hamartoma. Summary of patient characteristics and individual subject electrode coverage is provided in Supplementary Table 1. All patients provided individual informed consent as approved by the Stanford University Institutional Review Board (IRB #11354). This includes consent to publication of videos and demographics information.

### Statistics and reproducibility

This was a prospective human study between January 2020 and January 2021. The final sample size was based on clinical volume of our host institution, study duration, and patients meeting eligibility. No statistical method was used to predetermine sample size, however N equal or greater than three was required. As there was no intervention, blinding and randomization processes were not applicable to this study.

### Electrode registration and anatomical parcellation

Electrode location in 3D space was obtained from post-implant CT co-registered with the subject’s pre-operative MRI. Anatomic location of each contact was determined using FreeSurfer-based automated parcellation^50^. Electrodes in key limbic regions or outside of FreeSurfer’s anatomical catalog were validated by expert examination. For electrode visualization, the FreeSurfer average brain was used with coordinates in standard Montreal Neurologic Institute (MNI) space.

### Data acquisition and signal preprocessing

Using stereoelectroencephalography (sEEG), neural recording from implanted depth electrodes were sampled at 1024 Hz. sEEG preprocessing and analysis were performed using the FieldTrip^51^. First, a notch filter was used to attenuate power noise (60, 120, and 180 Hz), followed by a laplacian re-referencing scheme to minimize far-field volume conduction^52^. High-frequency activity (HFA, 70–170 Hz) was obtained by applying an 8th-order Butterworth bandpass filter and subsequently taking the absolute value of the Hilbert transform. This signal was boxcar smoothed using 60 s windows. Thus, the HFA at any given point represents an average of one-minute recording. Similarly, the analytic signals of delta (1–4 Hz), theta (4–8 Hz), alpha (8–12 Hz), beta (15–25 Hz), and gamma bands (25–70 Hz) were obtained using the same approach, except 4th order Butterworth filter was used as the bandpass filter. Interictal epileptiform discharges (IEDs) were computed using an automated algorithm as previously described^53^. IEDs were calculated in channels of the seizure onset zone (Subject 1) and in the probable seizure onset zone (Subject 3, channels with high IED rates given no electrographic seizures were captured). The seizure onset zone was defined by consensus from the epilepsy team. Continuous interstitial glucose level was obtained using the continuous glucose monitoring (CGM) system similar to that previously published for use in rodents^9^. The clinical CGM system implanted in the three subjects for the duration of the study was the Dexcom G6 model. The sensor was inserted into the abdominal subcutaneous tissue and sampled interstitial glucose levels at 5-min intervals. For every glucose measurement, the corresponding sEEG data was matched within ±1 s. Gaps in glucose measurements, at most three consecutive values, were interpolated using linear regression. Rate of change in glucose level was calculated as the discretized derivative of interstitial glucose levels over time. The use of CGM in ambulatory and inpatient setting have been previously described^20,49^.

### Discrete states identification

Video recording was used to identify four discrete states analyzed in this study. Ad libitum meals were defined as any substantial consumption of food items lasting at least 15 min. The meals were divided into pre-prandial (−3 to 0 h) and prandial (0 to 3 h) epochs. The index time (0 h) was at the time of meal initiation. Behavioral sleep periods were defined as periods of sustained eye closure without voluntary activity lasting at least 2 h. Any other periods were subsequently defined as wakeful periods. Automated sleep staging based on iEEG recordings was performed using the SleepSEEG algorithm^22^. For this analysis, we first downsampled our recordings to a sampling rate of 256 Hz. We then remontaged our recordings to a bipolar configuration, with adjacent contacts on the same electrode being subtracted from each other, as required by the SleepEEG algorithm. Finally, the SleepSEEG deep-learning model was applied to our dataset, excluding any electrodes that were determined to have epileptiform activity using their previously described automated detection algorithm^22^.

### Time-series correlation analysis

The lag-corrected correlation between continuous glucose measurements and sEEG signal was determined by first calculating the rectified cross-correlation function (XCF) within temporal lags of ±12 h and subsequently obtaining the Pearson’s correlation at the point of highest XCF (crosscorr.m, Econometrics Toolbox, Matlab). The chance level of correlation at each time lag was obtained by permutation testing whereby randomly shuffled 30-min segment of glucose measurements was correlated with the average gray matter sEEG signal. The time frame used for shuffling was chosen to maintain the temporal structure of the glucose time series, but small enough to allow numerous iterations of shuffling. This procedure was repeated for 1000 iterations. The chance correlation was defined at *p*-value of 0.05. To determine the lag-corrected correlation during discrete states (sleep, awake, pre-prandial, and prandial), glucose and sEEG timeseries were first corrected for temporal lag. Data indices associated with each discrete state were then applied to the lag-corrected timeseries, and Pearson’s correlation was subsequently obtained.

### Time-series wavelet analysis

The coherence between continuous glucose measurements and sEEG powerband were determined using Morlet wavelet coherence analysis (wcoherence.m, Wavelet Toolbox, Matlab)^17^. The coherence periodogram per powerband was obtained by averaging across the coherence spectrum across time. For regional analysis, the coherence values were calculated on a single channel basis first, then grouped together based on anatomy. The circadian coherence was defined as the mean coherence with periodicity of 24 h ± 33%, as to allow variation in periodicity^54^. A secondary analysis using a narrower range of 20–28 h as the definition of circadian rhythm was also performed. To determine the chance level of coherence for significance testing, we generated a null distribution whereby randomly shuffled glucose measurements at 30-min segments was used to compute coherence with the average gray matter sEEG signal for 1000 iterations. The chance coherence was defined at *p*-value of 0.05. To further characterize the timescale of coupling between continuous glucose measurements and sEEG powerband, the circadian component of the signal (24 h ± 33%) was removed using wavelet analysis to generate corresponding non-circadian (ultradian) signal (0–18 h) for both the continuous glucose and sEEG data^54^.

### Cortico-cortical evoked potential (CCEP) analysis

As part of the clinical seizure mapping, CCEPs were collected from Subject 1. We used CCEP mapping to identify regions that were functionally connected to the hypothalamus (i.e., exhibited a significant evoked potential after hypothalamus stimulation)^55–57^. Bipolar stimulation was performed across multiple limbic contacts including on bilateral hypothalamic leads. Approximately 50 (6 mA, biphasic) stimulation pulses were delivered at 1 Hz. CCEP analysis was subsequently performed offline. sEEG data was first preprocessed as described previously^56^. Briefly, the power line noise was removed, followed by removal of the stimulation artifact and common channel referencing. The preprocessed data was then epoched to 2 s in duration centered on the time of the stimulation pulse. The epoch data was baseline corrected to −150 to 50 ms. The CCEP was quantified by taking the mean signal between 10 and 150 ms. The initial 0 ms to 10 ms was not included to avoid roll-off effects from the stimulation artifact.

### Glucose decoder model construction, training, and evaluation

To investigate whether spectro-spatial profiles of intracranial electrophysiology can be used to predict interstitial glucose dynamics, we constructed a glucose decoder based on electrophysiologic features. Because of the large number of potential features present in the data (e.g., channels and powerband combinations), we employed LASSO (Least Absolute Shrinkage and Selection Operator) regression to minimize overfitting. The feature vector was constructed by first creating a N × P × t table, where N is the number of contacts, P is the number of powerbands (6 in this case), and t is the number of time points (sampled in 5-minute intervals). Next, the feature vector was flattened to a two-dimension 6N × t table, representing t different time points for model fitting. We split the time points randomly into a training set consisting of 80% of time points and a test set of the remaining 20%. Intuitively, LASSO regression selects for a subset of channels and powerbands that are best correlated with interstitial glucose and then uses multivariate linear regression for that selected subset to predict glucose levels. To ensure model stability, we use LASSO with Least Angle Regression (LASSO-LAR)^58^, as implemented in Python’s Scikit-learn package^59^. Briefly, LASSO regression takes a single parameter *α*, with higher values of *α* prioritizing selection of a smaller subset. In the LAR variant, parameters that are equally correlated to glucose levels, if selected, are both weighted equally in the model to ensure stability. To select an optimal *α*, we utilize 5-fold cross-validation, where we train a LASSO model for a certain *α* for 80% of the training set and test its performance on the remaining 20% of the data, with model performance defined by Pearson’s correlation between actual and predicted glucose levels. This process is repeated five times for a different 20% of the training set each time. We then repeat this process for different values of *α*, taking the α with the highest performance. We finally use the optimal α and train a LASSO model on the entire training set and evaluate performance with the test set. In Subject 2, limited time-points where interstitial glucose was greater or equal to 160 mg/dL were identified as times of hyperglycemia, and our model was trained to identify those time periods of hyperglycemia rather than provide an exact value given non-linear relationships observed between spectral activity and higher glucose levels.

### Determining glucose decoder circadian dependency

To determine whether glucose decoder performance is primarily attributable to only circadian autocorrelation or statistical features of our dataset, we performed several controls. First, we evaluated model performance for randomly shuffled blood glucose levels in 1-h segments, defined as the shuffled control. Second, we calculated the mean glucose level across all times points during a 24-h period. Correlation coefficients were then determined between the mean glucose levels and the measured glucose values to create the circadian control. In addition to quantifying the decoder performance for predicting actual glucose level, decoder performance was determined for decoding (1) the circadian component of the interstitial glucose dynamics and (2) deviations of the interstitial glucose levels from the average circadian glucose levels. Performance of the model against these two additional outputs provide an assessment of whether the model is better at predicting circadian or non-circadian (ultradian) dynamics.

### Identifying significantly used contacts in glucose decoder

As discussed above, LASSO regression only uses a subset of the most correlative channels and powerbands to decode interstitial glucose levels. To identify which regions of the brain are most frequently used in glucose decoding, a bootstrap Monte Carlo approach was utilized. Let *t* be the number of time points in our dataset. The glucose decoder was trained using data randomly drawn at time *t* over 1000 trials with replacement. For each training session, channels that were used in the decoder was recorded. Subsequently, an electrode was considered to significantly contribute to the glucose decoder if it was used at least 99% of the time. In addition, the median of the linear coefficients for each feature was reported, with the coefficient being zero if the electrode was not used.

### Reporting summary

Further information on research design is available in the Nature Portfolio Reporting Summary linked to this article.

## Supplementary information

Supplementary Information

Reporting Summary

## Data Availability

The data that support the findings of this study are available from the corresponding author on an individual request basis due to institutional data sharing agreements and compliance with U.S. Health Insurance Portability and Accountability Act (HIPAA). Source data are provided with this paper.

## Source data

Source Data

## Code availability

Matlab 2019b was used to perform all electrophysiologic analyses in this study. The dependent toolboxes used were: Fieldtrip-20190828, SPM8 (2013-09-17), SPM12 (2018-11-07), iELVis (2019-09-29), Matlab Signal Processing toolbox (r2019b). Python (>= 3.10) was used for decoding analysis and included these packages: h5py (>= 2.10.0), numpy (>= 1.19.2), scikit_learn (>= 1.1.2), scipy (>= 1.5.4). No new algorithm or preprocessing techniques were performed outside of standard toolbox usages. Customized code for glucose decoding is available on Github (https://github.com/dancingdarwin/CGM-Decoder?fbclid=IwAR2TIiA49gMZpq1CwjMF6oWD9jEw13tgk5yS-54yZAp-Kq7ihK79belUn3w).
