# Supplementary material for: Spectro-spatial features in distributed human intracranial activity proactively encode peripheral metabolic activity
Source: Nat Commun. 2023 May 11;14:2729. doi: 10.1038/s41467-023-38253-7 (PMC10174612; doi:10.1038/s41467-023-38253-7)
Supplement: Supplementary file 1 — Supplementary Information [file 41467_2023_38253_MOESM1_ESM.pdf]

Supplementary Materials for

Spectro-spatial features in distributed human intracranial activity proactively encode peripheral metabolic activity

Yuhao Huang*, Jeffrey B. Wang*, Jonathon J. Parker, Rajat Shivacharan, Rayhan A. Lal, Casey H. Halpern

Correspondence to: casey.halpern@gmail.com and inforay@stanford.edu

This PDF file includes:

Supplementary Figures 1 to S11
Supplementary Table S1

Supplementary Figures

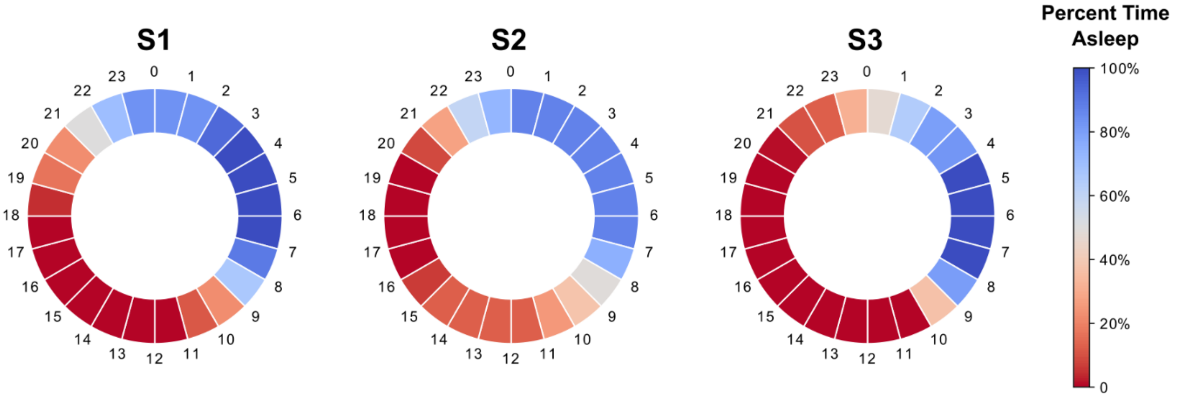

Supplementary Figure 1: Sleep chronotype shows distinct sleep/wake cycles for each study subject. Heat-map depicting sleep chronotype of subjects. Each slice represents 1 hour, and the color represents the percentage of days during the study for which the subject was asleep during that hour.

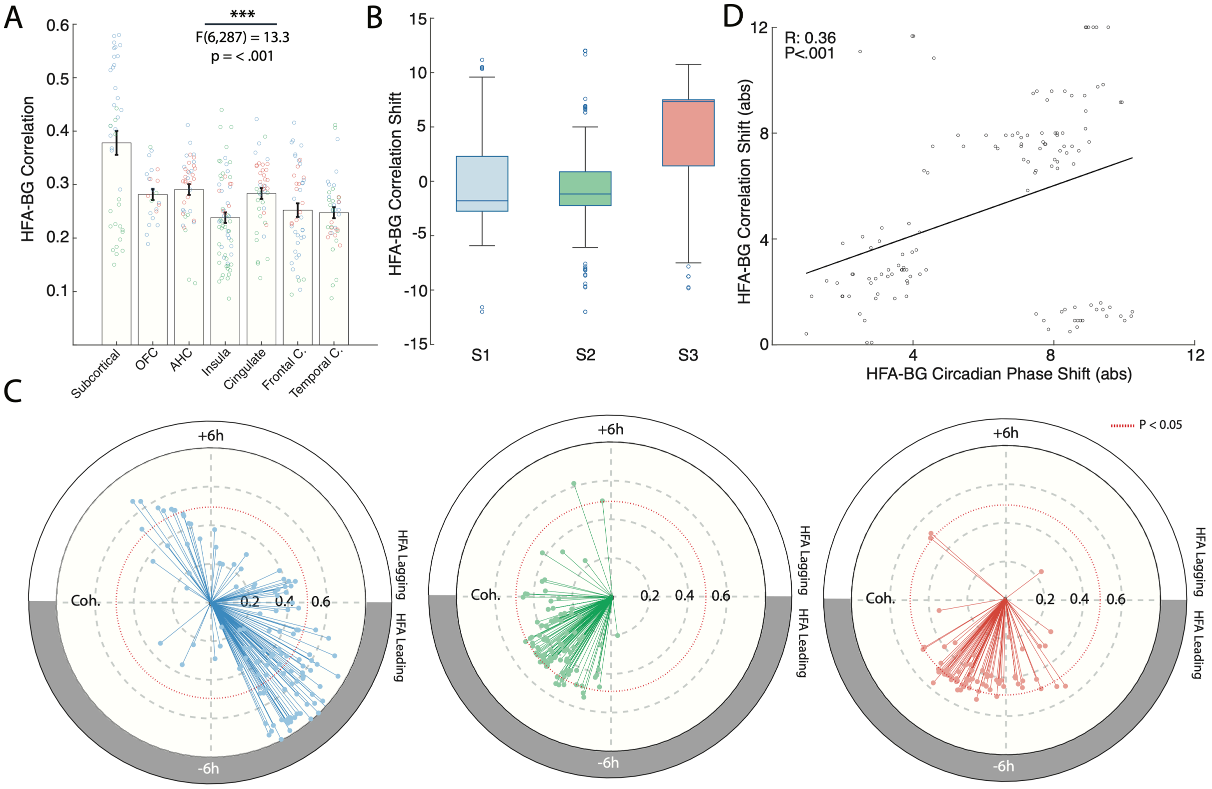

Supplementary Figure 2: Subcortical and corticolimbic HFA-glucose coupling and associated circadian coherence. (A) Lag-corrected correlation between HFA and interstitial glucose across subcortical and corticolimbic regions (Nsubj = 3, Nchannels = 288, One-Way ANOVA; F(6,287=13.3), P < .001). Error bars indicate standard error of the mean (SEM). **(B)** Distribution of correlation lag between HFA and interstitial glucose across 3 subjects (S1 maxima: 9.6 minima: -5.9, center: -1.79, 25% percentile: -2.8, 75% percentile: 2.3; S2 maxima: 5, minima: -6.1, center: -1.2, 25% percentile: -2.2, 75% percentile: 0.88; S3 maxima: 10.8, minima: -7.5, center: 7.3, 25% percentile: 1.4, 75% percentile: 7.5). **(C)** Left: phase diagram for Subject 1 showing the coherence and phase offset for all grey matter contacts. The mean circadian phase offset was 1.8 hours prior to the current glucose signal (Rayleigh's test, Z = 40.9, P < .001) with a mean coherence of 0.49. Middle: Phase diagram for Subject 2 showing the coherence and phase offset for all grey matter contacts. The mean circadian phase offset was 9.5 hours prior to the current glucose signal (Rayleigh's test, Z = 76.6, P < .001) with a mean coherence of 0.40. Right: Phase diagram for Subject 3 showing the coherence and phase offset for all grey matter contacts. The mean circadian phase offset was 7.3 hours prior to the current glucose signal (Rayleigh's test,

Z = 50.7, $P < .001$) with a mean coherence of 0.44. **(D)** Relationship between HFA-BG correlation temporal lag and HFA-BG circadian phase shift. The presence of significant correlation (Pearson's Correlation: $R = 0.36$, $P < .001$, Nchannels = 130) between the two time delay suggests that the circadian shift contributes to the temporal lag observed in the corrected HFA-BG correlation.

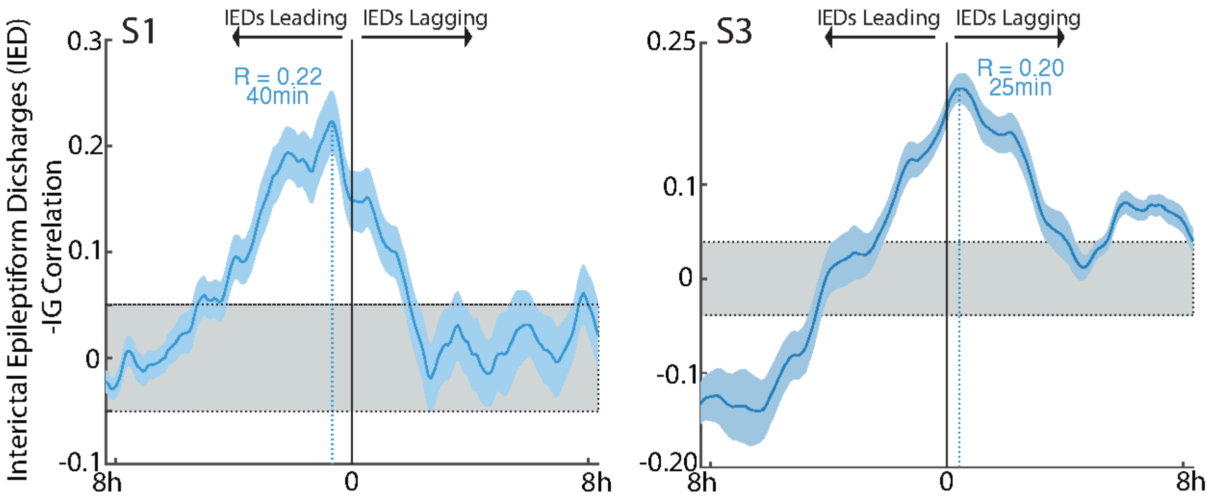

Supplementary Figure 3: Interictal epileptiform discharges (IEDs) show modest correlation with interstitial glucose variations. IEDs in the seizure onset zone and the probable seizure onset zone were computed for Subjects S1 (*left;* N = 4 electrodes) and S3 (*right*; N = 6 electrodes) respectively, as Subject S2 did not have epilepsy. The mean cross-correlogram between IEDs and interstitial glucose variations indicate significant but modest correlation when corrected for temporal lag. The approximated 95% confidence interval shown in gray is calculated for the cross-correlogram assuming the signals are uncorrelated. Shaded error bars indicate standard error of the mean (SEM).

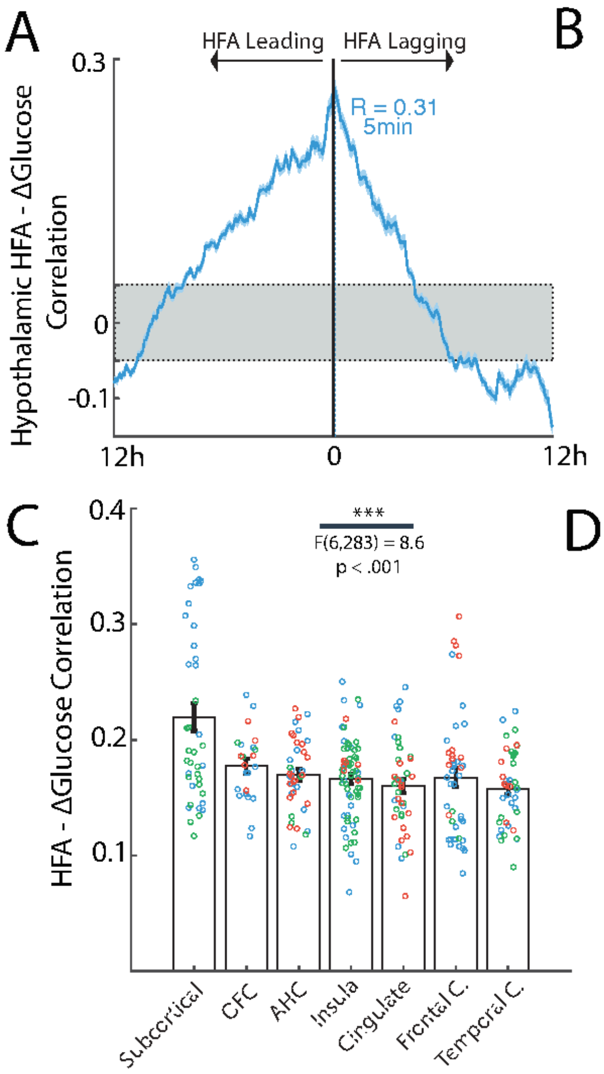

Supplementary Figure 4: Correlation of spectral activity in the hypothalamus and corticolimbic region with change in interstitial glucose levels. (A) Mean cross-correlogram between hypothalamic HFA and derivative of interstitial glucose variations. The error bar indicates the standard error across the hypothalamic contacts (N = 7). The approximated 95% confidence interval shown in gray is calculated for the cross-correlogram assuming the signals are uncorrelated. (**B)** Mean lag-corrected correlation between hypothalamic activity and derivative of interstitial glucose variations across conventional powerband (N = 7 hypothalamic channels, One-Way ANOVA; F(5,36=15), P < .001). **(C)** Lag-corrected correlation between HFA and derivative of interstitial glucose across subcortical and corticolimbic regions (Nsubj = 3, Nchannels = 288, One-Way ANOVA; F(6,283=8.6), P < .001). **(D)** Coherence periodogram between hypothalamic

HFA and derivative of interstitial glucose (N = 7 hypothalamic channels). Shaded error bars indicate standard error of the mean (SEM). All error bars indicate SEM.

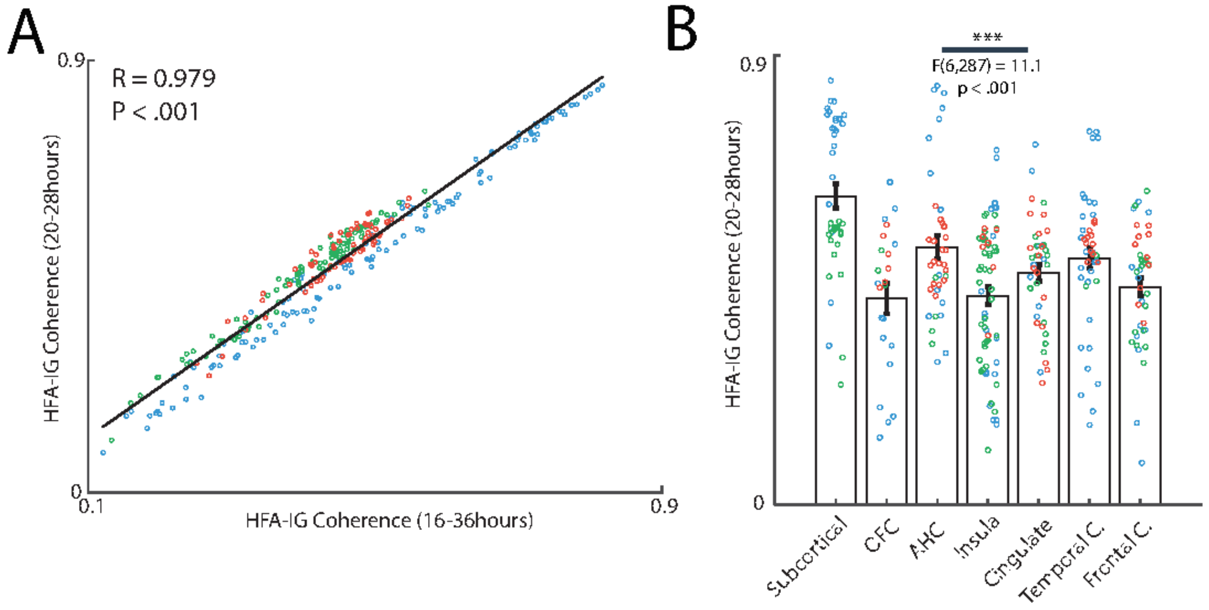

Supplementary Figure 5: Use of narrow circadian periodicity definition results in similar HFA-glucose circadian coherence. (A) Scatterplot of HFA-glucose circadian coherence computed using the 16-36 hour window and the 20-28 hour window. A strong correlation (Pearson's Correlation, R = 0.979, Nchannels = 294) between coherence computed with both windows was observed. **(B)** Circadian coherence computed using 20-28 hour periodicity between HFA and interstitial glucose across subcortical and corticolimbic regions (Nsubj = 3, Nchannels = 288, One-Way ANOVA; F(6,287=11.1), P < .001). All error bars reflect standard error of the mean (SEM).

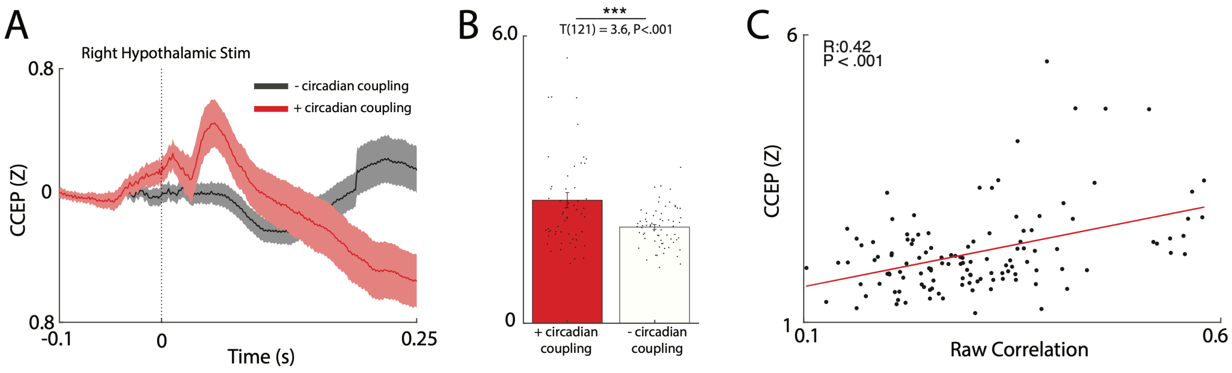

Supplementary Figure 6: Regional differences in HFA-Glucose coupling can also be partly explained by right hypothalamic connectivity. (A) Average cortico-cortical evoked potentials (CCEP) elicited by right hypothalamic stimulation in S1 stratified by whether channels' HFA were significantly coupled to glucose in a circadian manner. Channels with significant glucose coupling showed an overall larger CCEP with right hypothalamic stimulation. Shaded error bars indicate standard error of the mean (SEM). **(B)** Average CCEP magnitude (0.01 - 0.1s) was significantly higher in channels with significant circadian coherence to glucose (S1, Nchannels = 122, Two-Sample T-test: $t(121) = 3.6$, $P < 0.001$). Error bars indicate SEM. **(C)** Right hypothalamic CCEP magnitude correlates directly with raw correlation across channels in subject 1 (Pearson's $R = 0.42$, $P < .001$, Nchannels = 123).

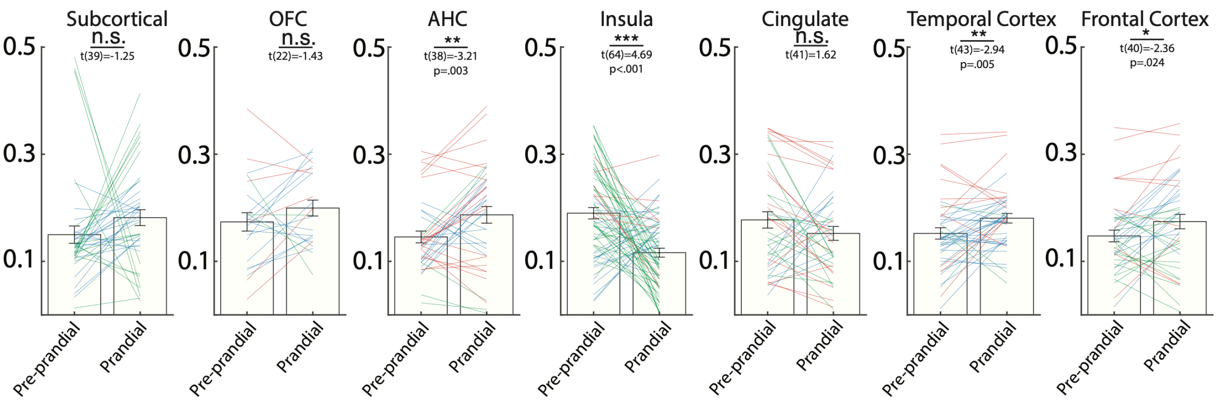

Supplementary Figure 7. Subcortical and corticolimbic lag-corrected non-circadian HFA-glucose correlation stratified by pre-prandial and prandial states across three subjects. Lag-corrected ultradian (non-circadian) correlation is decreased in the insula (Paired t-test: t(64)=4.7, P<.001) during prandial period while it is increased in the AHC and frontotemporal cortices (Paired t-test: all P<.001). Colors refers to the channels that belong to a particular subject. Blue: S1, Green: S2, Red: S3 (Nchannels per region is noted by the degree of freedom). All error bars reflect standard error of the mean (SEM).

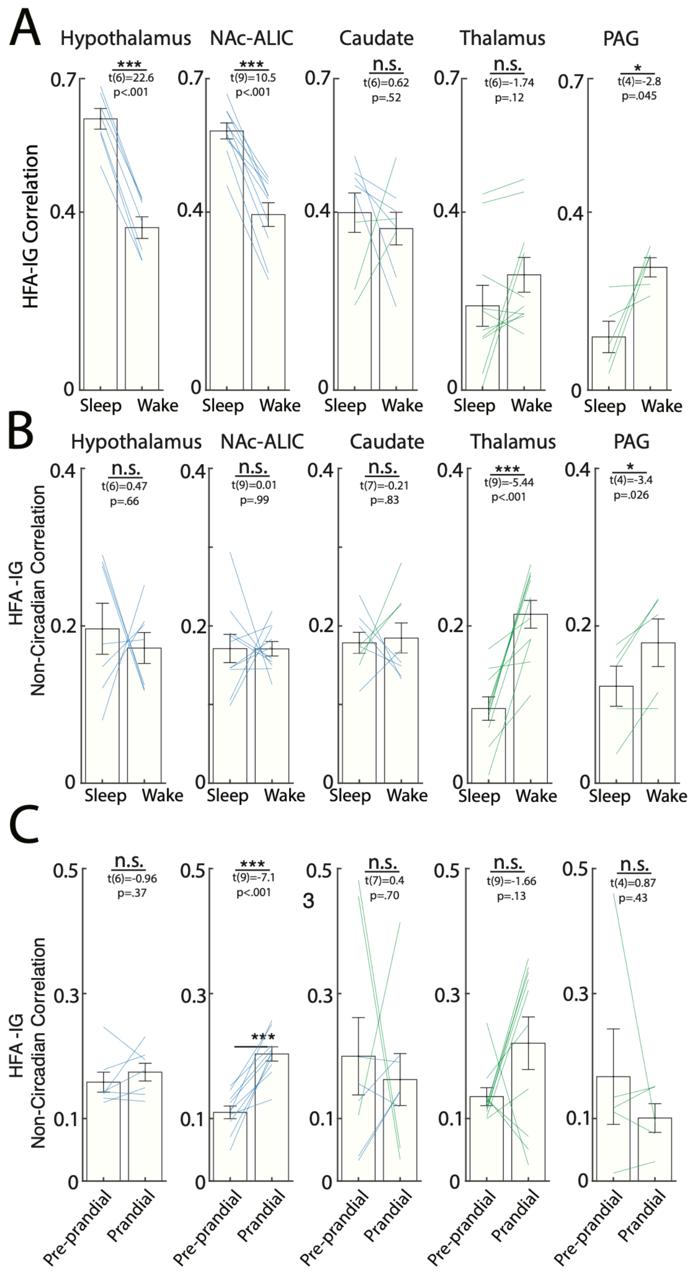

Supplementary Figure 8: Subcortical HFA-Glucose raw and non-circadian correlations as stratified by sleep, wake and meal-related states. (A) Subcortical lag-corrected overall HFA-glucose correlation stratified by sleep and wake states in Subject 1 and Subject 2 (Subject 3 did not have any subcortical coverage). **(B)** Subcortical lag-corrected non-circadian HFA-glucose correlation stratified by sleep and wake states. **(C)** Subcortical lag-corrected non-circadian HFA-glucose correlation stratified by pre-prandial and prandial states (Nchannels per region is noted by the degree of freedom, all tests were paired T-test with statistics and P-value shown in the figure). All error bars reflect standard error of the mean (SEM).

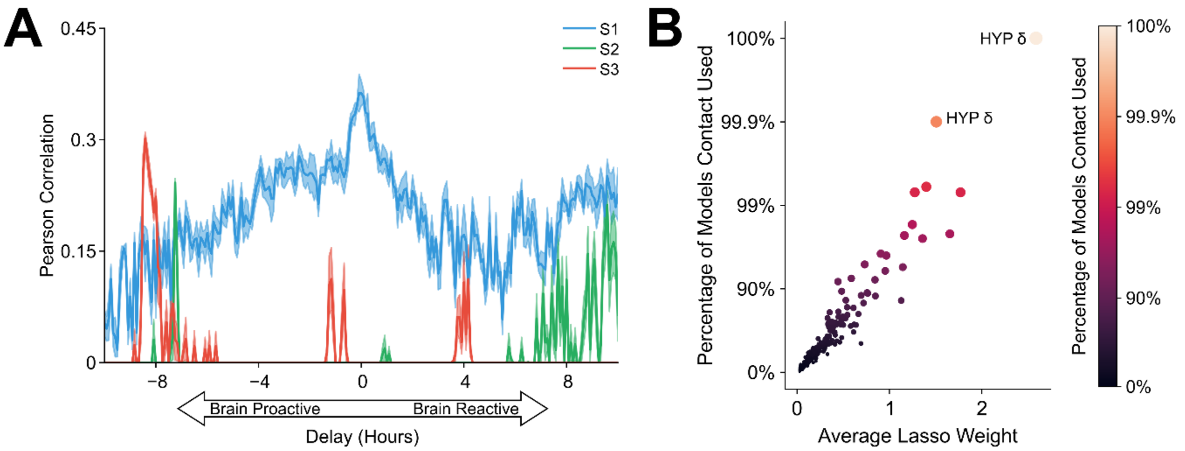

Supplementary Figure 9: Decoding derivative of interstitial glucose from intracranial spectral activity. **(A)** Model performance for decoding rectified derivative of interstitial glucose quantified by average Pearson Correlation between the actual and predicted interstitial glucose rate of change during 5-fold cross validation, as a function of different temporal lag between the iEEG spectral data and interstitial glucose. Negative shifts indicate current iEEG data predicts interstitial glucose in the future (i.e. proactive decoding). Shaded regions are ±1 SEM. **(B)** Scatterplot of model coefficients and how often LASSO selects this feature for Subject S1. Labeled datapoints appeared in at least 99% of models.

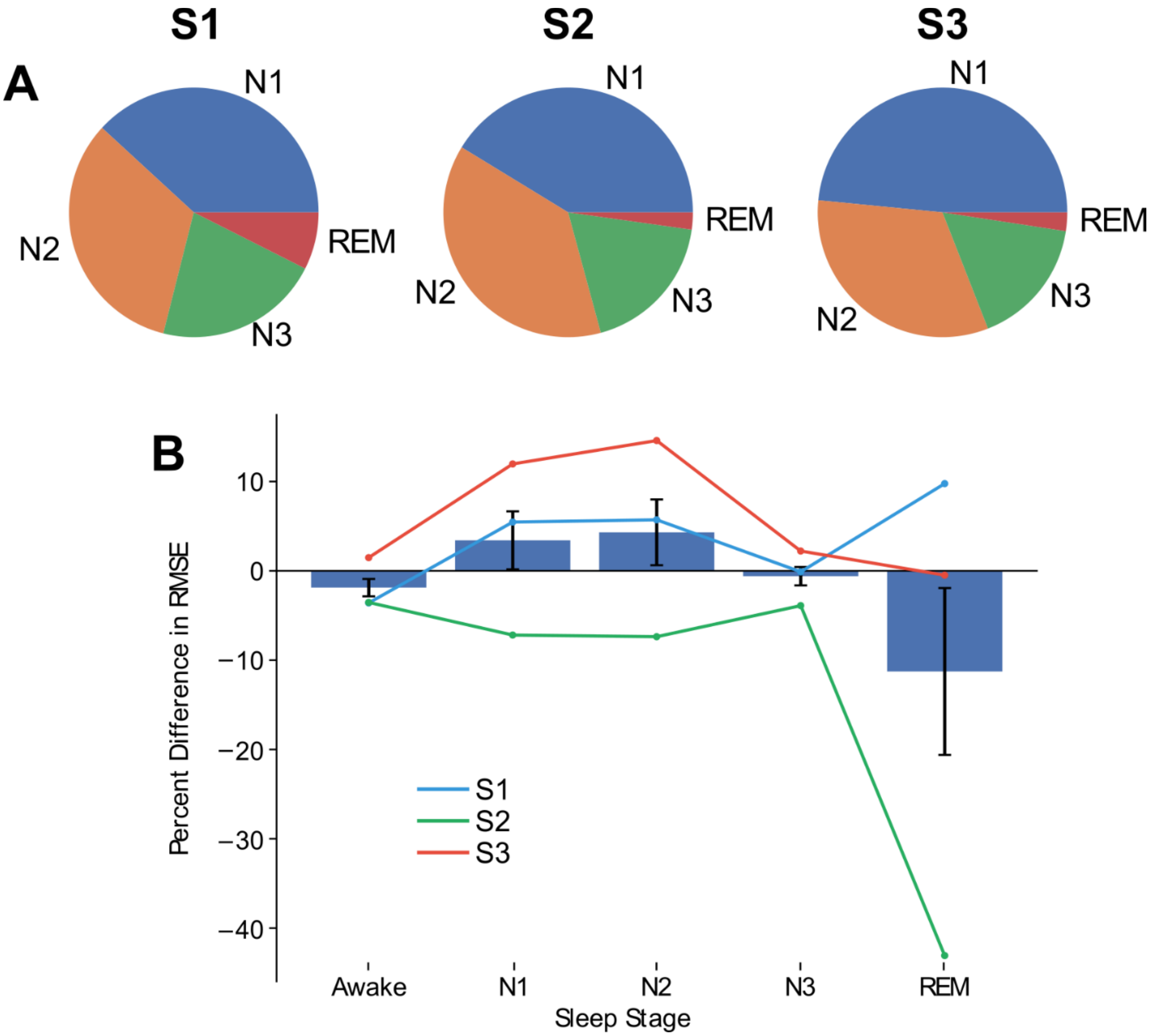

Supplementary Figure 10: Spectral decoding of interstitial glucose as stratified by sleep stages. Sleep stages were scored using the SleepEEG algorithm[25]. **(A)** Proportion of sleep spent in each sleep stage as scored by SleepSEEG. **(B)** Here we depict the change in Root Mean Square Error (RMSE) during each sleep stage. Error bars are +/- 1 SEM (N = 3 subjects).

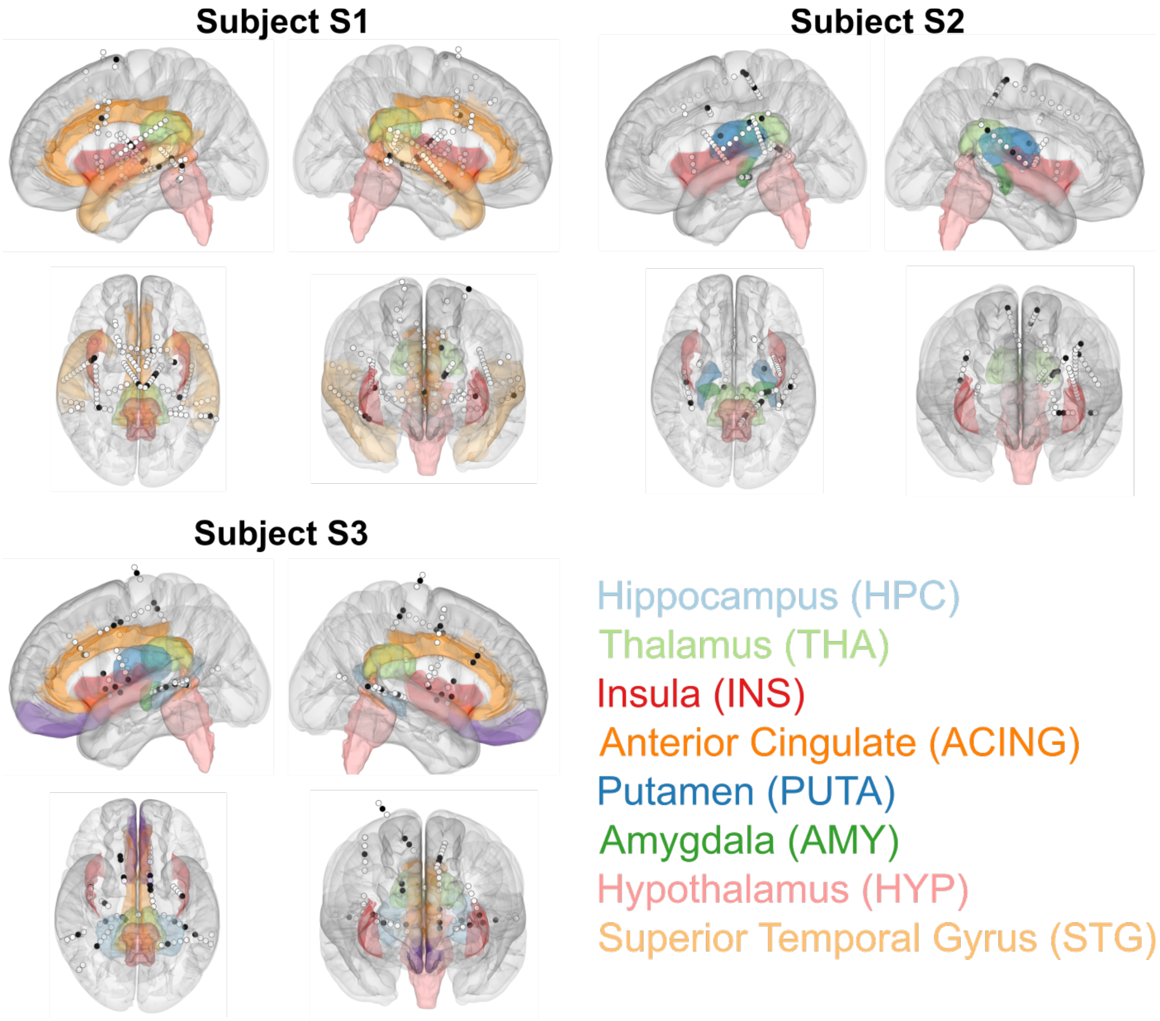

Supplementary Figure 11: Individual subject contacts used in glucose decoding model. White circles were used in less than 99% of LASSO models with randomized training and test sets while black circles were used in more than 99% of models.

Supplementary Table 1: Patient Characteristics and Coverage							
**ID**	**Age Range**	**Sex**	**BMI**	**Hgb A1C**	**Monitoring Indication**	**Recording Period**	**Coverage**
S1	20-30	F	37.5	5.2	Gelastic seizures. Seizure onset zone defined in right frontal heterotpia. Sz#1 (5:11:09AM to 5:12:18AM)<br>S#2 (11:26:27AM to 11:27:38AM)	5.5days	Hypothalamus (N = 7)<br>NAc-ALIC (N = 10)<br>Dorsal Striatum (N = 5)<br>OFC (N = 14)<br>AHC (N = 15)<br>Insula (N = 20)<br>Cingulate (N = 10)<br>Temporal Cortex (N = 29)<br>Frontal Cortex (N = 14)
S2	60-70	M	26.4	N/A	Intractable Pain Mapping*	6.5days	Dorsal Striatum (N = 3)<br>Thalamus (N = 10)<br>PAG (N = 5)<br>OFC (N = 3)<br>AHC (N = 5)<br>Insula (N = 38)<br>Cingulate (N = 15)<br>Temporal Cortex (N = 3)<br>Frontal Cortex (N = 17)
S3	50-60	F	29.1	6.0	R temporal seizures. No seizures captured but frequent discharges seen in posterior bilateral hippocampi	9.3days	OFC (N = 6)<br>AHC (N = 19)<br>Insula (N = 7)<br>Cingulate (N = 17)<br>Temporal Cortex (N = 12)<br>Frontal Cortex (N = 10)

NAc-ALIC: nucleus accumbens, anterior limb of internal capsule
OFC: orbitofrontal cortex
AHC: amygdalohippocampal complex
PAG: periaqueductal grey matter
*This patient was enrolled in an invasive monitoring protocol approved by Stanford Health Care's Innovative Care Committee to guide a future off-label deep brain stimulation surgery for refractory chronic pain which has yet to be established.
