# Supplementary material for: Spectro-spatial features in distributed human intracranial activity proactively encode peripheral metabolic activity
Source: Nat Commun. 2023 May 11;14:2729. doi: 10.1038/s41467-023-38253-7 (PMC10174612; doi:10.1038/s41467-023-38253-7)
Supplement: Supplementary file 2 — Reporting Summary [file 41467_2023_38253_MOESM2_ESM.pdf]

nature portfolio

Corresponding author(s): Casey Halpern
Ray Lal

Last updated by author(s): 03/08/2023

Reporting Summary

Nature Portfolio wishes to improve the reproducibility of the work that we publish. This form provides structure for consistency and transparency in reporting. For further information on Nature Portfolio policies, see our Editorial Policies and the Editorial Policy Checklist.

Statistics

For all statistical analyses, confirm that the following items are present in the figure legend, table legend, main text, or Methods section.

n/a	Confirmed	
☐	☒	The exact sample size (*n*) for each experimental group/condition, given as a discrete number and unit of measurement
☐	☒	A statement on whether measurements were taken from distinct samples or whether the same sample was measured repeatedly
☐	☒	The statistical test(s) used AND whether they are one- or two-sided<br>*Only common tests should be described solely by name; describe more complex techniques in the Methods section.*
☐	☒	A description of all covariates tested
☐	☒	A description of any assumptions or corrections, such as tests of normality and adjustment for multiple comparisons
☐	☒	A full description of the statistical parameters including central tendency (e.g. means) or other basic estimates (e.g. regression coefficient) AND variation (e.g. standard deviation) or associated estimates of uncertainty (e.g. confidence intervals)
☐	☒	For null hypothesis testing, the test statistic (e.g. *F*, *t*, *r*) with confidence intervals, effect sizes, degrees of freedom and *P* value noted<br>*Give P values as exact values whenever suitable.*
☐	☒	For Bayesian analysis, information on the choice of priors and Markov chain Monte Carlo settings
☐	☒	For hierarchical and complex designs, identification of the appropriate level for tests and full reporting of outcomes
☐	☒	Estimates of effect sizes (e.g. Cohen's *d*, Pearson's *r*), indicating how they were calculated

Our web collection on statistics for biologists contains articles on many of the points above.

Software and code

Policy information about availability of computer code

Data collection	Electrophysiologic data was obtained from intracranially placed depth electrodes (AdTech Medical) by using the clinically available Nihon Kohden EEG recording system. Continuous glucose data was obtained from Dexcom G6 continuous glucose monitoring devices.
Data analysis	Matlab 2019b was used to perform all electrophysiologic analyses in this study. The dependent toolboxes used were: Fieldtrip-20190828, SPM8 (2013-09-17), SPM12 (2018-11-07), iELVis (2019-09-29), Matlab Signal Processing toolbox (r2019b). Python (>= 3.10) was used for decoding analysis and included these packages: h5py (>= 2.10.0), numpy (>= 1.19.2), scikit_learn (>= 1.1.2), scipy (>= 1.5.4). No new algorithm or pre-processing techniques were performed outside of standard toolbox usages. Customized code for glucose decoding is available on Github (https://github.com/dancingdarwin/CGM-Decoder?fbclid=IwAR2TIiA49gMZpq1CwjMF6oWD9jEw13tgk5yS-54yZAp-Kq7ihK79belUn3w).

For manuscripts utilizing custom algorithms or software that are central to the research but not yet described in published literature, software must be made available to editors and reviewers. We strongly encourage code deposition in a community repository (e.g. GitHub). See the Nature Portfolio guidelines for submitting code & software for further information.

Data

Policy information about availability of data

All manuscripts must include a data availability statement. This statement should provide the following information, where applicable:

- Accession codes, unique identifiers, or web links for publicly available datasets
- A description of any restrictions on data availability
- For clinical datasets or third party data, please ensure that the statement adheres to our policy

The data that support the findings of this study are available from the corresponding author on an individual request basis due to institutional data sharing agreements and compliance with U.S. Health Insurance Portability and Accountability Act (HIPAA).

Human research participants

Policy information about studies involving human research participants and Sex and Gender in Research.

Reporting on sex and gender	Consecutively enrolled study participants were recorded for their demographic information including sex, which was individually included in the study.
Population characteristics	See behavioral & social sciences study design
Recruitment	All consecutive epilepsy patients undergoing seizure monitoring meeting the study eligibility between January 2020 to January 2021 were asked if they would like to participate in the study.
Ethics oversight	Stanford University Institutional Review Board (IRB #11354)

Note that full information on the approval of the study protocol must also be provided in the manuscript.

Field-specific reporting

Please select the one below that is the best fit for your research. If you are not sure, read the appropriate sections before making your selection.

☐ Life sciences ☒ Behavioural & social sciences ☐ Ecological, evolutionary & environmental sciences

For a reference copy of the document with all sections, see nature.com/documents/nr-reporting-summary-flat.pdf

Behavioural & social sciences study design

All studies must disclose on these points even when the disclosure is negative.

Study description	Three human subjects (two females) who were observed in an epilepsy monitoring unit for clinical seizure mapping met the inclusion criteria for this study from January 2020 to January 2021. This was an quantitative experimental study. The electrodes were placed for the sole purpose of clinical treatment and had no regard to this study. Patients also received a Dexcom G6 device for continuous glucose monitoring.
Research sample	3 consecutive human participants (2 female, 1 male, age 29-53) were enrolled. The inclusion criteria included 1) simultaneous coverage of hippocampus, amygdala, insula and orbitofrontal cortex (regions likely to harbor glucose-responsive neurons and are frequently targeted for epilepsy mapping), 2) no known history of diabetes mellitus and 3) at least 5 days of continuous monitoring. This was a consecutive series of patients meeting eligibility and represent the average patient seen in the Epilepsy Monitoring Unit. The chosen study sample provided a unique opportunity evaluate intracranial electrophysiology in addition to continuous peripheral glucose monitoring.
Sampling strategy	Consecutive enrollment of patients meeting the enrollment criteria (via convenience sampling). The final sample size was based on clinical volume of our host institution, study duration and patients meeting eligibility. No statistical method was used to predetermine sample size, however N of 3 was the minimum as this is a common threshold for studies of human intracranial electrophysiology.
Data collection	Nihon Kohden EEG-1200 and its supplied software was used for all video and EEG data capture in this study. The Dexcom G6 device was used to capture simultaneous glucose data. No blinding was performed in this study. The researchers instructed the participants on the tasks and operated the electrophysiology hardware and software. Family of the study subjects were occasionally present.
Timing	The subject enrollment period was from January 2020 to January 2021
Data exclusions	Subjects with lesions in the brain including mass, infarct and encephalomalacia were not enrolled in the study as a part of the pre-determined exclusion process.

Non-participation	No enrolled subjects declined to participate.
Randomization	No randomization process apply to this study as there was no intervention.

Reporting for specific materials, systems and methods

We require information from authors about some types of materials, experimental systems and methods used in many studies. Here, indicate whether each material, system or method listed is relevant to your study. If you are not sure if a list item applies to your research, read the appropriate section before selecting a response.

Materials & experimental systems

n/a	Involved in the study	
☒	☐	Antibodies
☒	☐	Eukaryotic cell lines
☒	☐	Palaeontology and archaeology
☒	☐	Animals and other organisms
☐	☒	Clinical data
☐	☒	Dual use research of concern

Methods

n/a	Involved in the study	
☒	☐	ChIP-seq
☒	☐	Flow cytometry
☒	☐	MRI-based neuroimaging

Clinical data

Policy information about clinical studies

All manuscripts should comply with the ICMJE guidelines for publication of clinical research and a completed CONSORT checklist must be included with all submissions.

Clinical trial registration	This study was not a clinical trial and thus not registered
Study protocol	Consecutive subjects meeting inclusion criteria were asked if they were interested in participating in this study during their hospitalization stay for intracranial seizure monitoring. No patients declined to participate which limits any self-selection bias. Subjects who has known lesions in their brain (encephalomalacia, mass, acute infarct) were excluded from enrollment as results from these subjects may be difficult to generalize to the broader population.
Data collection	The subject enrollment period was from January 2020 to January 2021
Outcomes	Quantitative associative measures between peripheral glucose recordings and intracranial brain recordings (correlation, coherence, decoding performance)

Dual use research of concern

Policy information about dual use research of concern

Hazards

Could the accidental, deliberate or reckless misuse of agents or technologies generated in the work, or the application of information presented in the manuscript, pose a threat to:

No	Yes	
☒	☐	Public health
☒	☐	National security
☒	☐	Crops and/or livestock
☒	☐	Ecosystems
☒	☐	Any other significant area

Experiments of concern

Does the work involve any of these experiments of concern:

No	Yes	
☒	☐	Demonstrate how to render a vaccine ineffective
☒	☐	Confer resistance to therapeutically useful antibiotics or antiviral agents
☒	☐	Enhance the virulence of a pathogen or render a nonpathogen virulent
☒	☐	Increase transmissibility of a pathogen
☒	☐	Alter the host range of a pathogen
☒	☐	Enable evasion of diagnostic/detection modalities
☒	☐	Enable the weaponization of a biological agent or toxin
☒	☐	Any other potentially harmful combination of experiments and agents
